# Salt Sensation and Regulation

**DOI:** 10.3390/metabo11030175

**Published:** 2021-03-17

**Authors:** Sonali Puri, Youngseok Lee

**Affiliations:** 1 Interdisciplinary Program for Bio-Health Convergence, Kookmin University, Seoul 02707, Korea; sonalibhu1201@gmail.com; 2 Department of Bio and Fermentation Convergence Technology, Kookmin University, Seoul 02707, Korea

**Keywords:** salt coding, taste perception, salt appetite

## Abstract

Taste sensation and regulation are highly conserved in insects and mammals. Research conducted over recent decades has yielded major advances in our understanding of the molecular mechanisms underlying the taste sensors for a variety of taste sensations and the processes underlying regulation of ingestion depending on our internal state. Salt (NaCl) is an essential ingested nutrient. The regulation of internal sodium concentrations for physiological processes, including neuronal activity, fluid volume, acid–base balance, and muscle contraction, are extremely important issues in animal health. Both mammals and flies detect low and high NaCl concentrations as attractive and aversive tastants, respectively. These attractive or aversive behaviors can be modulated by the internal nutrient state. However, the differential encoding of the tastes underlying low and high salt concentrations in the brain remain unclear. In this review, we discuss the current view of taste sensation and modulation in the brain with an emphasis on recent advances in this field. This work presents new questions that include but are not limited to, “How do the fly’s neuronal circuits process this complex salt code?” and “Why do high concentrations of salt induce a negative valence only when the need for salt is low?” A better understanding of regulation of salt homeostasis could improve our understanding of why our brains enjoy salty food so much.

## 1. Introduction

In addition to being one of the five basic senses, taste is also one of the two chemosensations, along with smell [1]. All life forms strive to consume integral nutrients and to steer clear from potential toxic supplements based on their unattractive taste. As an attempt to survive, numerous animals have developed chemoreceptors to determine deleterious chemicals by contact [2].

Both mammals and *Drosophila melanogaster* show mechanisms for coding the taste of salt. Salt is fundamental for the proper functioning of many physiological processes, including electrolyte homeostasis, muscle contraction, neuronal activity, and nutrient absorption. Although salt is key for the survival for mammals, excessive amounts of salt may cause hypertension and are therefore detrimental. Salt is perceived differently depending on the concentration, and is usually attractive below 100 mM and aversive above (the aversiveness intensifies as the concentration increases above this threshold) [3,4,5]. *Drosophila* is an influential model for functional analysis of the neuronal circuits behind behaviors. Although cardiovascular diseases, abnormal blood pressure, stroke, and hypertension resulting in death are highlight prevalent conditions associated with elevated levels of salt consumption [6], the actual cellular and molecular mechanisms in peripheral tissues and the brain by which high/low salt concentrations are encoded or recognized remain unclear, as is the effect of salt intake on consumption behavior. This review focuses on the studies of *Drosophila melanogaster* to determine the regulation of salt-sensing neurons in the brain and the effect of salt on physiological functions such as feeding behavior in mammals. The invaluable information obtained in these investigations will improve our understanding of the previously mentioned human diseases. Since all higher animals share innumerable functional and anatomical features of taste transduction, studies in *Drosophila* and mammals may also address our questions regarding sensory coding. Here, we present a critical and reliable examination of recent literature about salt sensation and regulation in mammals and *Drosophila* along with professional comment on prospective advancements.

## 2. Salt Taste in Mammals

**Figure 1:**
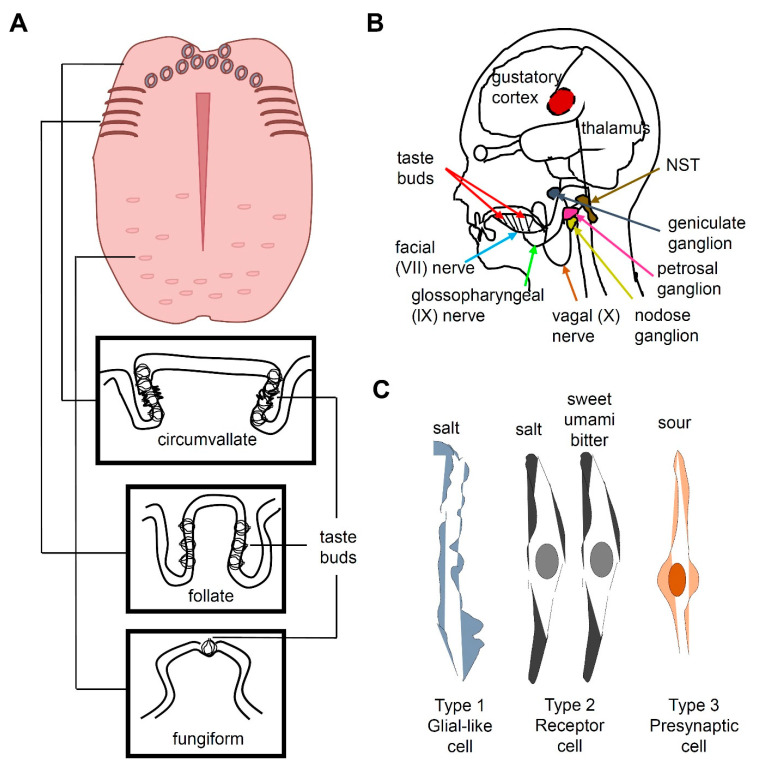
Taste perception in mammals. (**A**) Structure of a human tongue and the taste buds housing fungiform, folate, and circumvallate taste receptor cells (TRCs). (**B**) The neural pathway for human taste. The taste buds on the tongue and pharynx send gustatory information to the geniculate ganglion via the facial nerve VII, the petrosal ganglion via the glossopharyngeal nerve, and the nodose ganglion via the vagal nerve. This taste information is further transmitted to the thalamus and gustatory cortex via the nucleus of the solitary tract (NST) in the medulla oblongata. (**C**) Three morphologically distinct cell types (I, II, and III) are present in a taste bud and constitute five functional classes of sensory cells, each specialized to detect at least one of the five basic taste qualities. Type I TRCs are glial-like cells and earlier proposed to transduce an attractive salty taste, although it is controversial. Type II TRCs express T1R or T2R receptors and mediate the detection of sweet, umami, and bitter compounds. Distinct Type II TRCs from the sweet, umami, and bitter-sensing TRCs are salt sensitive. Type III TRCs, which are presynaptic cells, express otopetrin, a proton channel for sour taste.

Taste buds, which are ovoid structures composed of 50–100 taste receptor cells (TRCs), are located in the tongue and palate epithelium. Circumvallate and foliate papillae contain dense groupings of taste buds at the back or sides of the tongue, respectively, whereas fungiform papillae contain scattered taste buds at the front of the tongue (Figure 1A) [4,7]. TRCs are neuroepithelial cells innervated by pseudounipolar neurons whose cell bodies are located at the petrosal and geniculate ganglia (Figure 1B) [7]. Three morphologically distinct types of cells (I, II, and III) are present in a taste bud and constitute five functional classes of sensory cells, each specialized to detect at least one of the five basic taste qualities, namely, sweet, umami, bitter, salty, and sour (Figure 1C). Type I TRCs are glial-like cells and were earlier proposed to transduce an attractive salty taste [8,9] Type II TRCs express T1R or T2R receptors. Sweet and umami taste receptors are heterodimers of T1R2 + T1R3 and T1R1 + T1R3, respectively [10,11,12]. Bitter taste is mediated by T2Rs [7,13,14,15]. Type III TRCs express otopetrin, a proton channel mediating sour taste [16,17,18]. Type III TRCs also express PKD2L1 and carbonic anhydrase 4 [19,20,21]. However, the cell type associated with the perception of salty taste is controversial, because a recent study reported that sodium-taste cells can be considered as a Type II cell subset [9]. The salty Type II TRCs are distinct from sweet-, umami-, and bitter-sensing Type II TRCs and are characterized by the absence of *Trpm5* and presence of *ENaCα*. The cells dedicated to attractive sodium taste are positive for ENaC and CALHM1/3 [8].

Most of the taste-related information is transmitted via the facial nerve (VII) and the glossopharyngeal nerve (IX) (Figure 1B). The chorda tympani is a branch of the facial nerve that transmits taste messages from the anterior region of the tongue, which contains the fungiform papillae. The taste messages from the circumvallate papillae are transmitted via the glossopharyngeal nerve to the nucleus of the solitary tract (NST) in the medulla oblongata, while the foliate papillae send information via both VII and IX cranial nerves [22,23,24]. When the superficial petrosal and glossopharyngeal nerves are abolished, the chorda tympani nerve occupies the space in the terminal field, which indicates that these two nerves compete or prune axon terminals to occupy the terminal space during development [25]. The taste information in the NST passes to the thalamus, which projects to the cerebral cortex. The taste message is stored in the primary gustatory cortex in the insula and transported to the somatosensory cortex of the postcentral gyrus specifically dedicated to the tongue. This message is also carried to the prefrontal cortex, which coordinates taste association and perception of flavor. 

Salt responses are recorded from the chorda tympani nerve [18]. Depending on salt concentration, animals have two distinctive behaviors that are well reported in rodents: attraction (<100 mM) and aversion (>300 mM). At low concentrations, mice consume salt, but their behavior and neural responses are largely blocked by amiloride [26]. In contrast, at high concentrations of salt, mice exhibit innate aversion, and these responses are unaffected by amiloride. Thus, low concentration of salt is sensitive and high concentration of salt is insensitive to the diuretic amiloride [27]. Because epithelial Na^+^ channels (ENaCs) are specifically inhibited by amiloride, they have been proposed to participate in low-concentration salt taste [27,28]. ENaCs are non-voltage-gated and constitutively active channels that are highly selective of Na^+^ and Li^+^ over other monovalent cations such as K^+^ [27]. An ENaC is composed of three subunits [29], all of which are expressed in a small number of unique taste cells, with some being uncategorized TRCs [3]. In humans, the salt taste is amiloride-insensitive [30]. In addition to α, β, and γ ENaC subunits, humans express an additional δ ENaC subunit [31]. Heterologous expression of fully functional human α ENaC and δ ENaC was achieved in *Xenopus* oocytes [31,32,33]. This may provide the evidence that ENaC functions in salt sensation. Therefore, further studies are required to identify evolutionary changes in α, β, γ ENaCs in humans. ENaCs also play a major role in body fluid and blood pressure regulation [34]. The δ-ENaC is also expressed in the brain, pancreas, testis, and ovary. Therefore, it is interesting to find any other roles of ENaC in different organs. Amiloride-insensitive high-salt transduction constitutes ~80% of the high-salt responses recorded in the chorda tympani nerve in mice [35]. Furthermore, amiloride-insensitive high-salt responses are detected in type II TRCs, but not in type III TRCs in the fungiform papillae [36]. In type II TRCs, the salt response is highly dependent on the presence of Cl^-^, which is called “anion effect,” and involves the use of internal Ca^2+^ stores, whereas type III TRCs utilize voltage-gated Ca^2+^ channels activated by external Ca^2+^. Cl^−^, not Na^+^, plays a major role in the amiloride-insensitive high-salt responses [36], but the contribution of the cation remains unclear. In the circumvallate papilla, the amiloride-insensitive high salt response is mediated in type III TRCs through either an anion-selective or osmolality-sensitive channel [30,37]. Recent studies in the field of salt sensation have shown substantial progress in our understanding of the effects of low and high salt concentrations on peripheral responses in the tongue. Therefore, future studies in this regard should be focused on determining how excessive salt intake and hypertension can be avoided using the salt taste sensation.

## 3. Salt Taste in *Drosophila*

**Figure 2:**
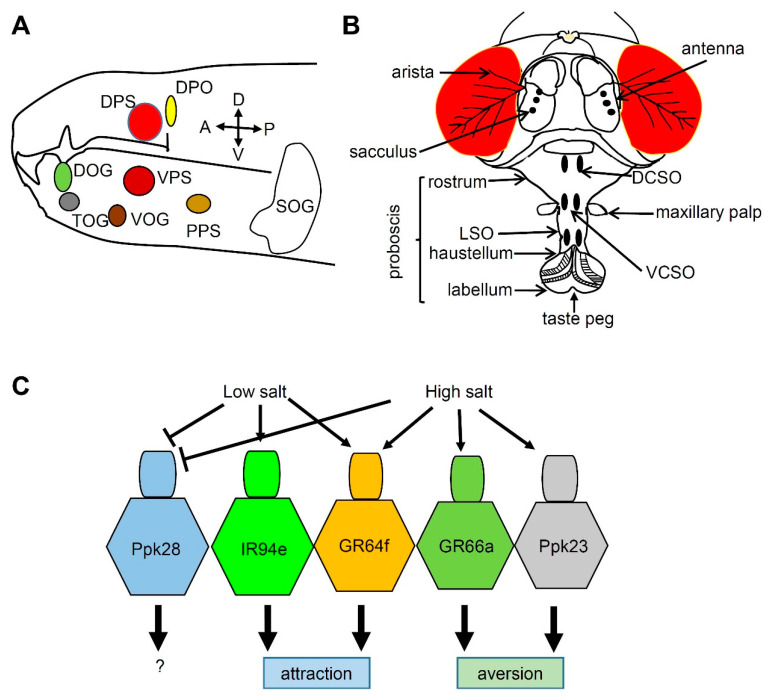
Chemosensory organs and taste sensation in *Drosophila melanogaster*. (**A**) The larval anterior part showing the chemosensory organs. DOG: dorsal organ ganglion, DPS: dorsal pharyngeal sensilla, DPO: dorsal pharyngeal organ, TOG: terminal organ ganglion, VOG: ventral organ ganglion, VPS: ventral pharyngeal sensilla, PPS: posterior pharyngeal sensilla. SOG (suboesophageal ganglion) indicates the first gustatory center in the brain. The orientation of the larvae is indicated by arrows. A: anterior, P: posterior, D: dorsal, V: ventral. (**B**) The adult fly head showing gustatory organs (labellum, taste peg, labial sense organ [LSO], dorsal cibarial sense organ [DCSO], and ventral cibarial sense organ [VCSO]) and olfactory organs (antenna and maxillary palp). Arista and sacculus sense temperature and humidity, respectively. (**C**) Fly gustatory receptor neurons (GRNs) can be divided into five classes based on the reporter expression. Low salt activates IR94e- and GR64f-positive GRNs, but inhibits water-sensing Ppk28 GRNs by osmolarity. High salt activates three types of GRNs, namely, GR64f-, GR66a-, and Ppk23-positive GRNs, but inhibits water-sensing Ppk28 GRNs.

The taste system in adult insects spreads throughout the body and includes the mouth, legs, wings, and ovipositor, where taste sensilla are located, although it is mainly distributed in the head region during the larval stage (Figure 2A) [38,39]. The mouth part is composed of the external organ, labellum, and the internal organ, the pharynx (Figure 2B). The 31 taste sensilla in the labellum are classified into long (L), intermediate (I), and short (S) bristles, depending on their length. Each sensillum contains 2–4 gustatory receptor neurons (GRNs).

Flies can sense five basic tastes—sweet, umami, bitter, salty, and sour—by utilizing chemoreceptors such as gustatory receptors (GRs), ionotropic receptors (IRs), transient receptor potential channels (TRPs), and pick pockets (PPKs) [40]. Flies enjoy ingesting low concentrations of salt but avoid high concentrations as humans do [41]. Among chemoreceptors, PPKs and IRs seem to meditate salt response in *Drosophila* during the larval and adult stages, respectively. Flies have 31 family members called PPKs, which are homologs of the amiloride-sensitive degenerin/epithelial Na^+^ channels (DEG/ENaC) [42]. Two PPKs, PPK11 and PPK19, detect Na^+^ and K^+^ during the larval stage [43,44]. However, it is not clear whether PPK11 and PPK19 are required for sensing salts during the adult stage. IR76b is required for attractive behavior to low salt concentrations [41], and *Ir76b* mutants have no electrophysiological response to low salt concentrations. IR25a and IR76b are also reported to play widespread roles in sensing low-salt, high-salt, and calcium levels, suggesting that IRs are major sensors that detect salt in insects [43,45,46]. Many genetic tools are available to express artificial reporters in flies by using the GAL4/UAS system, including GRN-specific reporters such as *Gr64f*-*GAL4* (sugar-sensing) [47], *Gr66a*-*GAL4* (bitter-sensing) [48], *ppk28*-*GAL4* (water-sensing, osmolarity sensitive) [49], *ppk23*-*GAL4* (calcium-sensing) [46], and *Ir94e*-*GAL4* (low salt-sensing) [43]. The cellular basis of salt-responsive behaviors was studied by expressing the Kir2.1 potassium channel in specific GRNs [43]. This study suggested that *Gr64f*-*GAL4-* and *Ir94e*-*GAL4-*expressing GRNs have low salt tuning properties and cause salt attraction, whereas *Gr66a*-*GAL4-* and *ppk23*-*GAL4-*expressing GRNs (especially the glutaminergic neurons of *ppk23^+^* GRNs) have high salt properties and mediate salt aversion (Figure 2C). However, the authors of that study did not test whether these GRNs mediate salt response during the larval stage. Furthermore, *Gr2a*-positive GRNs in the pharynx are involved in the aversion against high salt, although the *Gr2a* mutant show normal high-salt aversion [50]. This indicates that complex coding mechanisms to sense salt are separately mediated by the labellum and pharynx. Future studies should investigate the full repertoire of low- and high-salt sensors.

## 4. Regulation of Salt Homeostasis in Mammals

**Figure 3:**
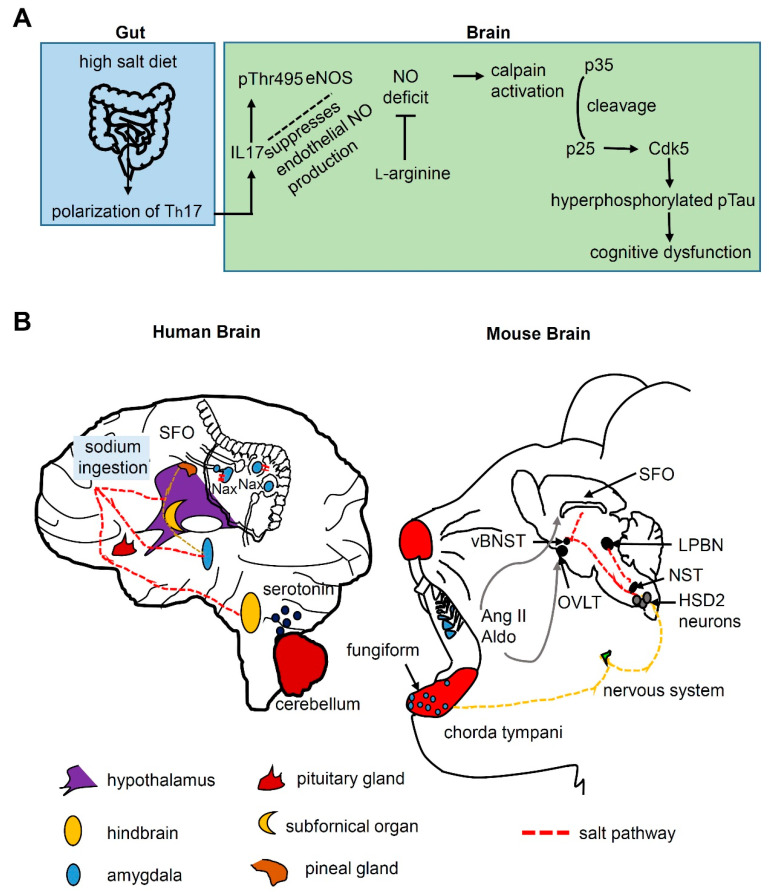
Salt appetite and regulation of salt homeostasis in the brain. (**A**) Gut-brain axis for a high salt diet. High dietary salt intake modulates T-helper 17 (Th17) and enhances the expression of the proinflammatory cytokine IL17, which suppresses endothelial NO production by inducing inhibitory phosphorylation of eNOS at Thr495. The NO deficit results in reduced calpain nitrosylation in neurons. This calpain activation cleaves p35 to p25, which activates Cdk5 and then phosphorylates Tau. The hyperphosphorylation of Tau is ultimately responsible for cognitive dysfunction. Rescue of NO deficit with l-arginine prevents the cognitive dysfunction. (**B**) The salt appetite-related signaling pathway in human and mouse brains. Sodium ingestion is regulated by three regions as indicated by the dotted lines in the human brain. The serotonin receptor in LPBN suppresses salt appetite. The Na_x_ channel is a salt sensor expressed in glial cells surrounding the subfornical organ (SFO). The taste messages in the mouse are processed to the lateral parabrachial (LPBN) via the NST. Aldosterone-sensitive NTS HSD2 neurons projecting to the medial central lateral parabrachial nucleus (PBN), the pre-locus coeruleus (pre-LC) and the ventral bed nucleus of the stria terminalis (vBNST) increase sodium intake. Two populations of neurons in the SFO project to the organum vasculosum of the lamina terminalis (OVLT) and vBNST to increase water and sodium intake, respectively. Angiotensin II (Ang II) and aldosterone (Aldo) influence the SFO and the OVLT.

The results of experiments in which mouse brains were altered by high-salt diets imply that such a diet may cause cognitive defects through accumulation of proteins instead of changes in blood flow (Figure 3A) [51,52]. On the one hand, mice on high-salt diets show a reduction in NO. NO relaxes blood vessels, thus decreasing the blood flow in the brain. However, this reduction in blood flow is insufficient to have a noticeable impact on cognitive abilities. On the other hand, extremely high-salt diets increase the levels of the proinflammatory cytokine IL17 via T-helper 17 (Th17) cells in the gut. The enhanced IL17 expression inhibits endothelial NO production by inducing eNOS phosphorylation. Reduction of NO levels in the brain results in reduced calpain nitrosylation, which cleaves p35 to p25. This activates Cdk5 and phosphorylates Tau. The fact that these clumps of Tau are known to be linked with dementias, including Alzheimer’s disease, leads to the idea that a cognitive reduction is triggered by Tau accumulation. Therefore, it is suspected that clumps of Tau made cognitive tasks such as recognizing objects and navigation difficult for mice on high-salt diets. This was further supported by the fact that mice on a high-salt diet supplemented with a compound that enhances NO production showed lower rates of Tau accumulation, indicating that decreased NO rates instead of blood flow alterations were responsible for the effects of high salt concentrations on Tau phosphorylation. The key point is that although mice fed a high-salt diet experience reduced brain blood flow, the phosphorylated Tau is responsible for the drop in intelligence (Figure 3A) [51,52].

Sodium taste sensation appears to be vital for the regulation of sodium appetite. Sodium appetite can be suppressed by the brain to prevent homeostatic deviations of salt balance. Various sites in the brain regulate sodium ingestion, including the hypothalamus, amygdala, and hindbrain (Figure 3B) [53]. The amygdala is also one of the brain areas transmitting information regarding sweet and bitter tastes [54]. The basolateral amygdala is the target of sweet cortex projections, while the central amygdala is the target of bitter cortex projections. The subfornical organ (SFO), the circumventricular organ (CVO) on the ventral surface of the fornix, is the major location for control of salt ingestion, wherein the Na_x_ channel is a salt sensor that is expressed in glial cells surrounding the sensory CVOs in the brain [55]. The Na_x_ channel is sensitive to sodium concentration, but not to voltage. The GABAergic neurons in the SFO show increased action potentials on application of hypertonic Na^+^. Through cholecystokinin-induced activation of GABAergic neurons, thirst-driving neurons can be suppressed under sodium-depleted conditions [56]. Two neuronal populations of SFO AT1a convey increase of water and sodium intake to the organum vasculosum of the lamina terminalis (OVLT) and the ventral bed nucleus of the stria terminalis (vBNST), respectively [57]. These distinct mechanisms in the SFO underlie the selective intake of water or salt. Therefore, they contribute to body fluid homeostasis [55,58]. Furthermore, the neural circuits and molecular mechanisms regulating sodium appetite have been reported (Figure 3B) [59,60]. Using pharmacological approaches, serotonin 2c receptor (Htr2c)-expressing neurons in the lateral parabrachial nucleus (LPBN) have been shown to suppress sodium appetite. LPBN is located at the junction of the pons and midbrain. The caudal solitary tract sends information to the LPBN, which transmits signals to the medial hypothalamus. Lateral parabrachial lesions failed to increase the sodium appetite even during euvolemic conditions [61]. This may be caused by the heterogeneous nature of the LPBN because this nucleus also contains a population of neurons that may promote sodium appetite [62].

Salt appetite, an instinctive state that drives animals to crave consumables containing sodium, may be triggered by aldosterone, a physiologic steroid hormone derived from the zona glomerulosa that regulates the stability of salt and water and preserves sodium in the distal renal tubule [63]. Adrenal glands are activated by sodium deficiency and secrete aldosterone partly independent of glucocorticoids and angiotensin II stimulation. Angiotensin causes vasoconstriction and an increase in blood pressure through the renin-angiotensin system. It also induces aldosterone release from the adrenal cortex to promote sodium retention. Neuronal activation of 11β-hydroxysteroid dehydrogenase type 2 (HSD2) increases sodium appetite, because they are sensitive to aldosterone [59]. HSD2 neurons project to the medial central lateral parabrachial nucleus (PBN), the pre-locus coeruleus, and the vBNST [59]. These putative circuits for salt appetite should be verified in the future.

## 5. Regulation of Salt Homeostasis in *Drosophila*

Salt is a powerful agent that enhances food intake in animals. The mechanism underlying salt perception in the brain and promotion of salt ingestion is not well understood. The nutritional requirement for sodium in adult humans is approximately 2.5 g/day [64,65]. Using powerful genetic tools and mutant libraries of *D. melanogaster*, age-related regulation of salt homeostasis, salt-related behavioral changes, and cellular and molecular basis of salt sensation in the brain can be studied. The findings of these studies will improve our understanding of the mechanisms in much more complex organisms, including humans. Like numerous species of the animal kingdom, *Drosophila* show signs of strong craving for salt during reproduction, probably because mating changes the way sensory neurons respond to the taste of salt. Unlike other post-mating behaviors such as yeast appetite, the salt appetite does not require octopamine, which suggests a divergent circuitry to regulate the post-mating behavior [66]. During reproduction, female flies grow a greater need for sodium, which changes pursuant to their diet. This new diet with increased salt intake promotes reproduction of flies and other animals. This salt appetite is not influenced by the salt essentials of egg-laying but by a feed-forward alteration of taste, triggered in a female body by a signal acting on post-mating circuitry originally from a male. Sex peptides travel through specific sensorimotor systems. Adult fly-feeding behavior is upregulated at 0.1 M salt and downregulated at 0.4 M salt. The greatest effect on salt appetitive behavior is observed between 0.05 M and 0.1 M salt concentrations [67]. These observations follow the results regarding larva adequately enough to allow us to suspect certain commonalities in the practical purpose of salt between both life stages of a fly [68].

A study of a *Drosophila* mutant that responds to aversive concentrations of salt but not to appetitive concentrations opens up novel ideas to regulate hypertension and high blood pressure in humans. SLC5A11, which belongs to a sodium/solute co-transporter (SLC5A) family (consisting of members structurally similar to the SLC5A proteins of humans, such as monocarboxylate, iodide, and multivitamin co-transporters for transport in the intestinal and renal lumen), plays a critical role in nutrient selection independent of the taste. Although sodium/glucose co-transporters of humans (SGLTs) have a distinct clade based on protein alignment in a phylogenic tree, they show approximately 24–30% amino acid identity to the *Drosophila* SLC5As [69,70]. SLC5A11 is prominently expressed in 10–13 pairs of R4 neurons of the ellipsoid body in the brain and functions to select appropriate foods. A reduction in the salt concentration of the hemolymph may trigger corresponding food-choosing behaviors. Although SLC5A11 may perform likewise (its mutation interrupting the transportation of salt), this may influence the choice between low and high levels of salt, damaging food choice independent of taste. The ellipsoid body R4 neurons that harbor SLC5A11 may be the cellular substrate that evaluates nutritional value by direct activation in relation to nutritive sugar during the prandial increase of sugar levels in the hemolymph [69,70]. Thus, the mechanism by which SLC5A11 moderates salt consumption would be an intriguing topic to study.

## 6. Salt-Inducible Kinase

First spotted in the adrenal glands of mice on high-salt diets, the role of salt-inducible kinase (SIK) in human illnesses, particularly tumorigenesis and diabetes, has been rarely investigated [71]. SIKs self-phosphorylate and play a role in regulating adrenocortical function under the stimulation of high salt levels or adreno-corticotropic-hormone (ACTH). Since members of the SIK family are dysregulated in several cancers, including, prostate, ovarian, breast, and lung cancers, SIKs are suspected to play a critical role in the formation and development of tumors. SIKs are evolutionary conserved, with *Drosophila melanogaster* showing SIK homologs termed dSIK and Kin-29 [72]. In humans, SIK1 is produced copiously in the adrenal cortex and adipose and neural tissues, while SIK2 and SIK3 are predominantly produced in the adipose tissues and are omnipresent in neural tissues [73,74,75]. SIK3 acts as a positive mediator for the inflammatory signal response that leads to cancer cell proliferation caused by high salt intake [76]. Calcium influx, triggered by sodium intake and affected by the exchange system of Na^+^/Ca^2+^(NCE1), could lead to SIK1 phosphorylation, and the activation induced by Ca^2+^/calmodulin-dependent protein kinase (CaMK) [77,78,79] on stimulation by sodium homeostasis can indicatively activate SIKs [80].

In *Drosophila*, both SIK2 and SIK3 act as upstream regulators in the Hippo signaling pathway, which is a pathway to control organ size by regulating cell proliferation and apoptosis [81,82]. By directly phosphorylating the scaffold protein Salvador (Sav, a crucial component of the Hippo complex), SIK2 and SIK3 can disturb the oncogene-driven inhibition of Yorkie (Yki), an ortholog of Yes-associated protein (YAP) [81]. As an important hub of the Hippo signaling pathway, YAP activation facilitates tumor cell metastasis in addition to inhibiting cell contact [83]. The Hippo signaling pathway was conservative and initially found in *Drosophila*, and was said to control organ size determination [83]. SIK2 and SIK3 are designated as negative regulators in Hippo signaling. The effect of Sik2/3 on Hippo signaling is partially mediated by Sav phosphorylation on Ser-413. Since SIK is linked to nutrient sensing, a relationship between systemic growth control and the Hippo pathway has been presumed to exist. SIKs are suspected to be potential oncogenes in lung and ovarian cancers. Thus, SIK2 inhibitors may serve as the stimulus for Hippo pathway activity in ovarian tumor cells [73,81,84,85].

Flies show biphasic behavior depending on temporal aspect (morning and evening) mediated by molecular oscillation in pacemaker neurons [86]. The SIK3-HDAC4 regulatory pathway allows the morning cells, the critical circadian pacemaker neurons of the fly brain to regulate specific downstream circadian neurons and behaviors [87]. To influence the synchronization of clock neurons, PDF up-regulates cAMP, actuates PKA, and modulates the balance of the genes Period (PER) and Timeless (TIM) in PDF receptor (PDFR)-expressing target neurons. PDF is an indirect circadian regulator of SIK3 activity via PKA. Based on the known functions of SIK, salt intake may contribute to cell and organ growth, tumorigenesis, and circadian regulation. However, it is not known whether wild-type flies and SIK3 mutants show any different salt-ingesting behaviors at different times of the day.

## 7. Conclusions and Future Perspectives

Taste receptors were first characterized in mammals as GPCRs, but insects mostly use ionotropic receptors including GRs, TRPs, IRs, and PPKs. For salt tasting, ENaCs (PPKs in *Drosophila*) and IRs are not GPCRs, but ionotropic and very sensitive to sodium concentrations. These receptors show maximal sensitivity to low concentrations of salt. However, previous studies did not clarify the types of sensors for high salt concentrations in mammals and *Drosophila*, which is an important question from the perspective of preventing hypertension in humans. Furthermore, our understanding of the process of adaptation to a new salt appetite remains limited. For example, the set-point for salt can be easily adapted to the amount of salt ingested over several days, indicating the presence of a critical mechanism to reset the salt appetite and related neural circuits. Identification of new molecular sensors for salt and the related neural controls such as hormones, neuropeptides, and neurotransmitters may yield insights into the coordination of processes in the nervous system.
